# Au-Based Bimetallic Catalysts for Aerobic Oxidation of 5-Hydroxymethylfurfural to 2,5-Furandicarboxylic Acid under Base-Free Reaction Conditions

**DOI:** 10.3390/molecules29122724

**Published:** 2024-06-07

**Authors:** Juan Su, Zongyang Liu, Yuan Tan, Yan Xiao, Nannan Zhan, Yunjie Ding

**Affiliations:** 1 Hangzhou Institute of Advanced Studies, Zhejiang Normal University, 1108 Gengwen Road, Hangzhou 311231, China; sujuan@dicp.ac.cn (J.S.); liuzongyang@zjnu.edu.cn (Z.L.); xiaoyan@dicp.ac.cn (Y.X.); nnzhan@zjnu.edu.cn (N.Z.); 2 Key Laboratory of the Ministry of Education for Advanced Catalysis Materials, Zhejiang Normal University, 688 Yingbin Road, Jinhua 321004, China; 3 Dalian National Laboratory for Clean Energy, Dalian Institute of Chemical Physics, Chinese Academy of Sciences, 457 Zhongshan Road, Dalian 116023, China; 4 The State Key Laboratory of Catalysis, Dalian Institute of Chemical Physics, Chinese Academy of Sciences, 457 Zhongshan Road, Dalian 116023, China

**Keywords:** gold catalyst, bimetallic catalysis, HMF oxidation, base-free, FDCA

## Abstract

The aerobic oxidation of 5-hydroxymethylfurfural (HMF) to 2,5-furandicarboxylic acid (FDCA) plays a pivotal role in the synthesis of renewable, biodegradable plastics and sustainable chemicals. Although supported gold nanoclusters (NCs) exhibit significant potential in this process, they often suffer from low selectivity. To address this challenge, a series of gold-M (M means Ni, Fe, Cu, and Pd) bimetallic NCs catalysts were designed and synthesized to facilitate the selective oxidation of HMF to FDCA. Our findings indicate that the introduction of doped metals, particularly Ni and Pd, not only improves the reaction rates for HMF tandem oxidation but also promotes high yields of FDCA. Various characterizations techniques, including X-ray diffraction (XRD), transmission electron microscopy (TEM), X-ray photoelectron spectroscopy (XPS), in situ diffuse reflectance infrared Fourier transform spectroscopy of CO adsorption (CO-DRIFTS), and temperature-programmed desorption of oxygen (O_2_-TPD), were employed to scrutinize the structural and electronic properties of the prepared catalysts. Notably, an electronic effect was observed across the Au-based bimetallic catalysts, facilitating the activation of reactant molecules and enhancing the catalytic performance. This study provides valuable insights into the alloy effects, aiding in the development of highly efficient Au-based bimetallic catalysts for biomass conversions.

## 1. Introduction

**Scheme 1:**
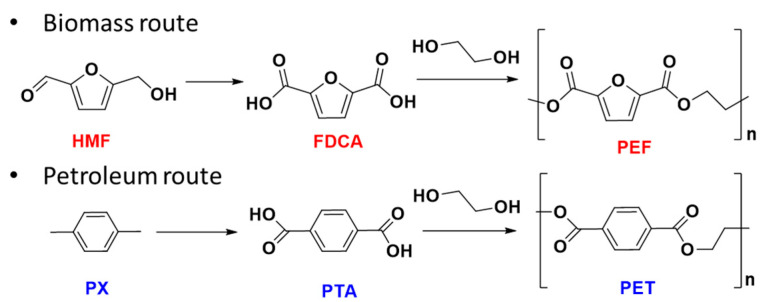
The synthetic routes for PEF and its counterpart PET.

The synthesis of fuels and chemicals from renewable resources, particularly biomass, has spawned significant attention recently in response to the depletion of fossil feedstocks and escalating environmental concerns [1,2,3,4]. 5-Hydroxymethylfurfural (HMF), a crucial biomass platform molecule, is derived from lignose and cellulose-based carbohydrates, making it a promising intermediate for the synthesis of various chemicals and alternative fuels within the bio-refinery framework [5,6,7,8]. One noteworthy application of HMF is the selective oxidation to form 2,5-furandicarboxylic acid (FDCA) (Scheme 1), which has attracted considerable interest due to its potential to serve as a key monomer in the production of renewable, biodegradable plastics, like polyethylene furandicarboxylate (PEF), a sustainable alternative to petroleum-derived polyethylene terephthalate (PET) [9,10,11].

Heterogeneous catalysts, particularly those containing precious metals such as Pt [12,13], Pd [14,15], Ru [16,17], and Au [18,19,20,21], have been extensively investigated for the catalytic oxidation of HMF to FDCA, utilizing air or O_2_ as the oxidant. However, the current systems face challenges related to low product selectivity and catalyst stability [17,22]. Additionally, obstacles such as high oxygen pressure, ion leaching, and the requirement for a high ratio of homogeneous base (1~20 equiv. NaOH) need to be overcome [13,21]. Supported Au catalysts have shown significant potential in this process. Au-based catalysts demonstrate greater stability and selectivity for the catalytic oxidation of HMF to FDCA in water compared to conventional precious metals [23,24]. Nevertheless, Au nanoparticles are susceptible to aggregation, leading to deactivation, particularly in the absence of a base promoter [25,26]. Therefore, synthesizing stable Au catalysts for the oxidation of HMF, especially under base-free conditions, presents a substantial challenge and remains an area of intense interest. 

Thiolate-protected ultrasmall gold nanoclusters (NCs) have emerged as important precursors for synthesizing supported gold catalysts with controllable sizes and exceptional performance [27,28,29]. Due to their precisely defined nanostructures and strong quantum size effects, these Au NCs exhibit significant potential across various fields such as catalysis, energy conversion, and biomedicine [30,31,32,33]. Among the different types of NCs, Au-based alloy NCs have garnered particular interest in catalytic processes due to their synergistic effects and superior properties compared to their monometallic counterparts [34,35]. By incorporating additional metals through doping and alloying, it is feasible to effectively modulate the optical, structural, and electronic properties of Au NCs [14,36]. For instance, the integration of Pd into Au NCs (Au-Pd NCs) has been shown to enhance their activity and stability in aerobic alcohol oxidation, surpassing that of pure Au NCs [37], which facilitates electron transfer from Pd to Au, thereby modulating the electronic structure. Similarly, bimetallic Au-Cu NCs have demonstrated heightened activity and stability in Ullmann C–O coupling reactions, outperforming both monometallic Au and Cu NCs [38,39]. What is noteworthy is that a recent study from our group demonstrated that Ag-doped Au NCs display significantly enhanced catalytic performance and catalyst stability in the aerobic oxidation of HMF. This enhancement was attributed to a synergistic effect between Ag and Au NCs [40]. However, despite these significant findings, a comprehensive investigation into the impact of metal doping on the oxidative reactions of Au catalysts still lacks sufficient evidence.

The research detailed in this study focuses on the design and synthesis of a series of transition metal-doped Au NCs for the aerobic oxidation of HMF to FDCA. Our approach harnesses the unique properties of thiolate-protected ultrasmall Au NCs and their alloy counterparts, which exhibit enhanced control over size and improved catalytic performance in comparison with the traditional gold nanoparticles. Hydrotalcite (HT), a commonly used solid base support, was employed to disperse the Au and bimetallic Au-M NCs, thereby obviating the necessity for additional inorganic alkali solution [41,42]. The results demonstrated that the Au-based bimetallic NCs exhibited enhanced activity and selectivity in comparison to the monometallic Au catalyst. To elucidate the underlying reasons responsible for this improved performance, various characterization techniques, such as X-ray diffraction (XRD), transmission electron microscopy (TEM), X-ray photoelectron spectroscopy (XPS), and in situ diffuse reflectance infrared Fourier transform spectroscopy of CO adsorption (CO-DRIFTS), were employed to analyze the morphology and structure of the catalysts. It was observed that the presence of different metal dopants induced alterations in the electronic structure of the gold catalyst, subsequently influencing the catalytic performance of the various metal-doped gold catalysts. Overall, this work underscores the significance of bimetallic catalyst synthesis and provides valuable insights into the application of alloy catalysts for sustainable chemical processes.

## 2. Results

### 2.1. Characterization of the Catalysts

**Figure 1:**
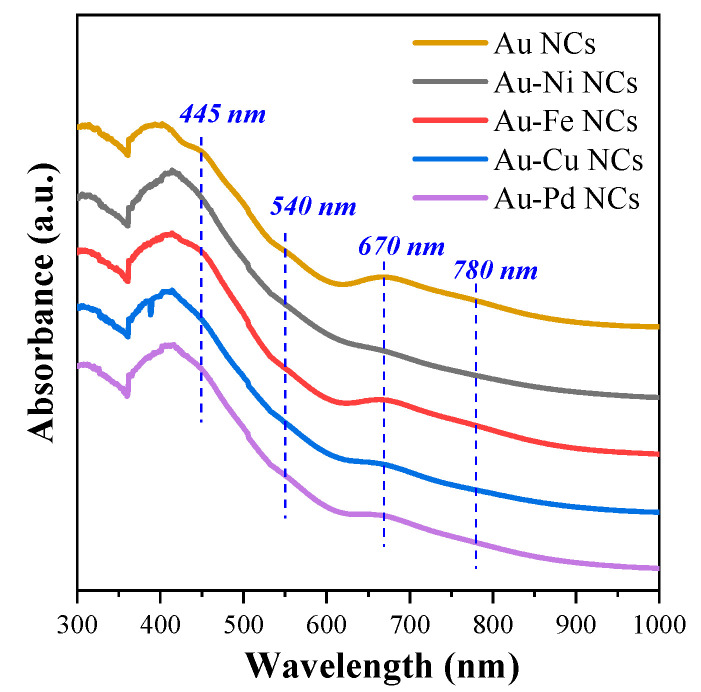
UV–vis spectra of Au_25_ and Au-M (M = Ni, Fe, Cu, and Pd) bimetallic NCs.

**Scheme 2:**
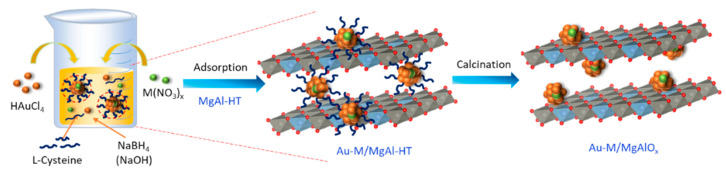
Schematic illustration for the synthesis of supported Au-M bimetallic catalysts.

In this study, four types of Au-based bimetallic nanoclusters (Au-M NCs, M = Ni, Fe, Cu, and Pd) were synthesized using L-cysteine as the protective ligands and NaBH4 as the reductant, with the Au/M molar ratios being constant at 20. For comparison, the monometallic gold nanoclusters were also synthesized via the same preparation process. The composition of the clusters was determined through UV–visible spectroscopy, in which four fingerprint peaks positioned at 445, 540, 670, and 780 nm were observed (as seen in Figure 1), which signified the successful preparation of “magic Au clusters” with 25 Au atoms [43,44]. Notably, the doping of trace metal atoms into Au NCs did not significantly affect the characteristic absorption peaks of Au25, revealing the successful preparation of well-defined Au-M bimetallic NCs in this work. Subsequently, the fresh synthesized Au-M bimetallic NCs and monometallic Au NCs were impregnated onto the support of MgAl-hydrotalcite (HT), followed by a drying and calcination process, to produce the final catalysts. The synthesis procedure is depicted in Scheme 2.

**Figure 2:**
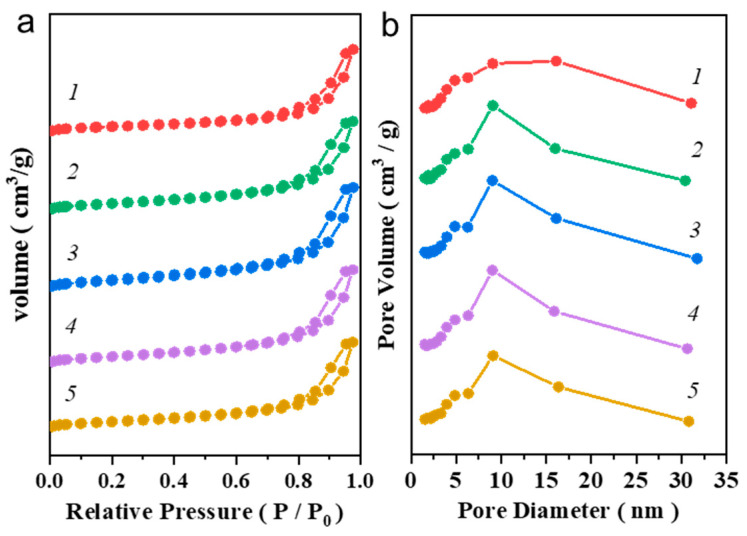
(**a**) N_2_-physisorption isotherms and (**b**) BJH-pore size distributions of the supported Au and Au-M bimetallic catalysts: (1) Au-MgAlO_x_; (2) Au-Ni/MgAlO_x_; (3) Au-Fe/MgAlO_x_; (4) Au-Cu/MgAlO_x_; and (5) Au-Pd/MgAlO_x_.

The obtained Au-M (M = Ni, Fe, Cu, and Pd) bimetallic catalysts and monometallic Au catalysts were characterized by inductively coupled plasma atomic emission spectroscopy (ICP-AES) to determine their elemental composition. The analysis data showed that the gold content was constant at approximately 1.1 wt.% in all catalysts, while the content of the additive metals (Ni, Fe, Cu, and Pd) ranged from 0.018 to 0.031 wt.%. The molar ratios of Au/M were calculated to be 18, 15, 14, and 19 for the Au-Ni, Au-Fe, Au-Cu, and Au-Pd samples, respectively, which is basically consistent with the theoretical value of 20. Additionally, from N2 physisorption isotherms and pore size distribution curves (as presented in Table 1 and Figure 2), all the samples exhibited a similar specific surface area (SBET), total volume, and pore sizes, indicating that the alteration of the additive metals did not obviously change the pore structure of catalysts.

**Figure 3:**
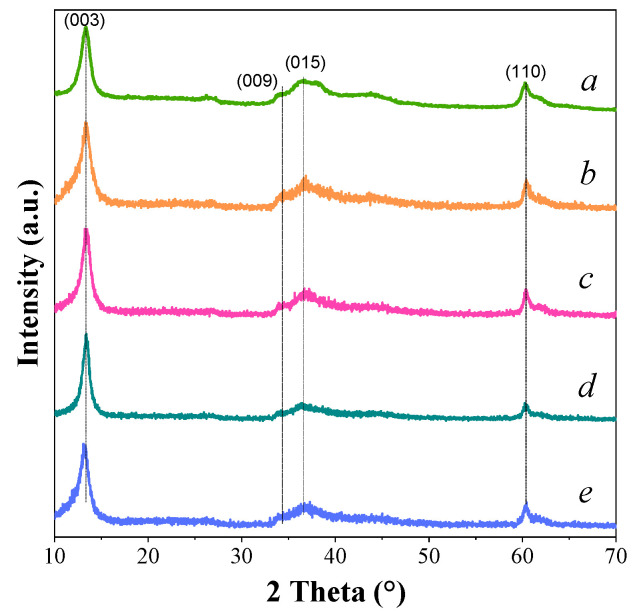
XRD patterns of different supported Au-based catalysts: (a) Au/MgAlO_x_; (b) Au-Ni/MgAlO_x_; (c) Au-Fe/MgAlO_x_; (d) Au-Cu/MgAlO_x_; and (e) Au-Pd/MgAlO_x_.

X-ray diffraction (XRD) analyses were conducted to examine the crystal phase of the above catalysts. In Figure 3, all catalysts exhibit diffraction peaks at 13.5°, 34.6°, 38.8°, and 60.5°, corresponding to the (003), (009), (015), and (110) planes of the hydrotalcite-derived MgAlO_x_ [45,46,47]. Notably, no discernible diffraction peaks attributable to Au or other metals can be observed in the spectra, indicating the highly dispersed nature of Au and Au-M NCs on the catalysts [48]. These results collectively suggest that the presence of doped metals does not alter the primary phase of the catalysts.

**Figure 4:**
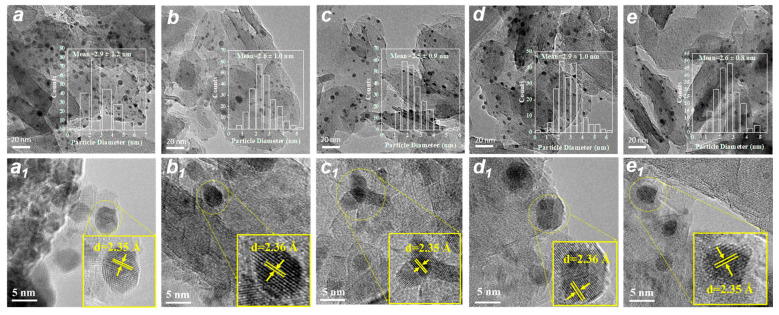
TEM and HRTEM images of the supported Au and Au-based bimetallic catalysts: (**a**,**a_1_**) Au/MgAlO_x_; (**b**,**b_1_**) Au-Ni/MgAlO_x_; (**c**,**c_1_**) Au-Fe/MgAlO_x_; (**d**,**d_1_**) Au-Cu/MgAlO_x_; and (**e**,**e_1_**) Au-Pd/MgAlO_x_.

The use of transmission electron microscopy (TEM) allowed for the examination of the dispersion of Au and Au-M NCs and the measurement of their average particle sizes. The TEM images in Figure 4a~e suggested that Au and Au-M NC were highly dispersed on the surface of all catalysts. The mean particle sizes were calculated to be 2.9 ± 1.2 nm for Au/MgAlOx (Figure 4a), 2.6 ± 1.0 nm for Au-Ni/MgAlOx (Figure 4b), 2.5 ± 0.9 nm for Au-Fe/MgAlOx (Figure 4c), 2.9 ± 1.0 nm for Au-Cu/MgAlOx (Figure 4d), and 2.6 ± 0.8 nm for Au-Pd/MgAlOx (Figure 4e). Moreover, by performing high-resolution TEM analysis (Figure 4(a1~e1)) and studying the lattice parameter of the particles, it can be observed that the Au (111) crystal faces were present in all samples [49]. The interplanar crystal spacing was approximately 2.35 Å, indicating that the incorporation of the doped metals did not significantly alter the basic geometry of the Au particles. Notably, given the low concentration of doped metals in the bimetallic nanoparticles, it is difficult to ascertain their exact structural configuration from the TEM images. Further characterization techniques, such as X-ray absorption fine structure (XAFS) analysis, might provide deeper insights into the structural characteristics of these bimetallic nanoparticles.

To investigate the chemical state and surface composition of the above catalysts, X-ray photoelectron spectroscopy (XPS) analysis was conducted. The XPS survey of all catalysts is presented in Figure S1, revealing elements such as Au, Mg, Al, C, O, etc. The surface atomic ratios of these elements in the above catalysts are detailed in Table S1, showing that Mg, Al, and O are predominant on the surface, which is attributed to the MgAl-HT-derived mixed metal oxides. Additionally, the presence of Au, S, and various doped metals is evident in the Au and Au-based bimetallic catalysts, signifying the successful preparation of thiol-protected Au and Au-based bimetallic catalysts. However, the surface atomic ratio of the doped metals, particularly Cu and Ni, is extremely low, making it challenging to detect their signal in specific regions of the spectrum (see Figure S2). These findings are supported by the results obtained from the ICP-AES analysis (refer to Table 1).

**Figure 5:**
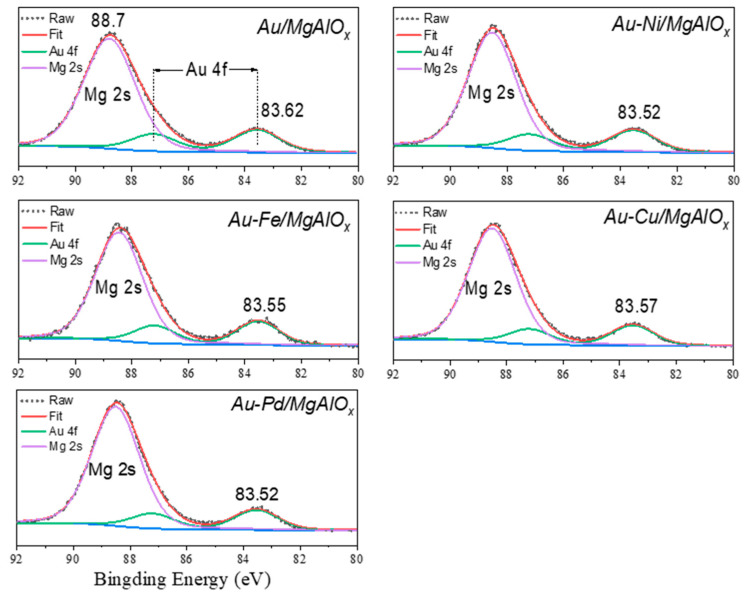
Au 4f XPS spectra of the Au and Au-based bimetallic catalysts.

Regarding Au, Figure 5 illustrates the binding energies (B.E.) of the Au 4f spectra, calibrated using the C 1s peak at 285 eV as the internal standard. The XPS peaks of Au 4f_7/2_ and Au 4f_5/2_ XPS are observed at approximately 83.62 and 87.29 eV, respectively, indicating the presence of metallic gold (Au^0^) [15,50]. Although the Au 4f_5/2_ band partially overlaps with the Mg 2s XPS peak located at 88.7 eV, a clear distinction of the electronic state of Au is still achievable through deconvolution of the Au 4f_7/2_ band. Notably, the B.E. of Au 4f_7/2_ for Au-Ni/MgAlO_x_ (83.52 eV), Au-Fe/MgAlO_x_ (83.55 eV), Au-Cu/MgAlO_x_ (83.57 eV), and Au-Pd/MgAlO_x_ (83.52 eV) shifts to lower values compared to Au/MgAlO_x_ (83.62 eV), indicating the presence of negatively charged gold species over the Au-M bimetallic catalysts, possibly resulting from the electron transfer from the doped metal to gold [16,51,52].

**Figure 6:**
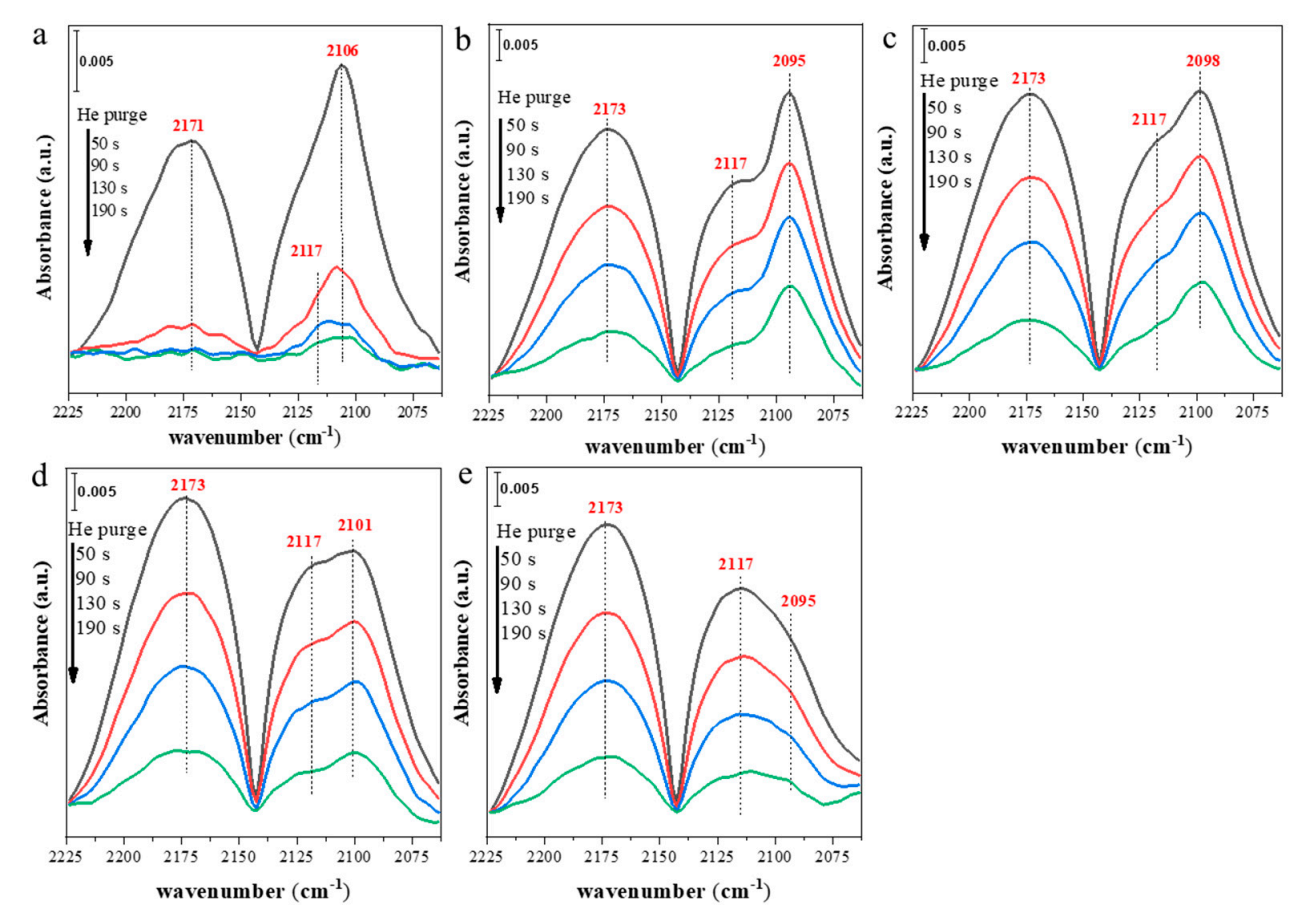
In situ CO DRIFT spectra of the Au and Au-based bimetallic catalysts: (**a**) Au/MgAlOx; (**b**) Au−Ni/MgAlOx; (**c**) Au−Fe/MgAlOx; (**d**) Au−Cu/MgAlOx; and (**e**) Au−Pd/MgAlOx. The black, red, green, and blue lines represent He blowing for 50 s, 90 s, 130 s, and 190 s, respectively.

In situ diffuse reflectance infrared Fourier transform spectroscopy of CO adsorption (CO-DRIFTS) serves as another effective method for probing the chemical states of metal sites. Figure 6 displays the result of in situ CO-DRIFTS analysis of the supported Au and Au-M bimetallic catalysts, revealing prominent CO adsorption bands at 2100, 2117, and 2173 cm^−1^, respectively. The double bands at 2117 and 2173 cm^−1^ are indicative of gas phase CO adsorption [53], with their peak intensity gradually decreasing upon helium purging. In the case of Au/MgAlO_x_, an additional peak positioned at 2106 cm^−1^ suggests the presence of CO molecules chemisorbed on metallic gold species [54,55]. Interestingly, upon the introduction of other metals to the catalyst, CO adsorption bands exhibit a red-shift towards lower wavenumbers. Specifically, for the Au-Ni/MgAlO_x_ and Au-Fe/MgAlO_x_, a sharp and strong peak at 2095 and 2098 cm^−1^ are observed. In addition, Au-Cu/MgAlO_x_ and Au-Pd/MgAlO_x_ display weak shoulder peaks at 2101 and 2095 cm^−1^, respectively. The higher wavenumber for the CO adsorption bands suggests a lower electron density on the gold surface [56], indicating that the introduction of doped metals enhances the electron density of gold. This speculation is supported by the XPS results. 

### 2.2. Catalytic Performances of HMF Oxidation to FDCA

The catalytic performances of HMF oxidation to FDCA were evaluated using supported Au and Au-M bimetallic catalysts in base-free reaction conditions within a batch-type stainless steel autoclave. The results are presented in Table 2. Analysis of the data reveals that the monometallic Au catalyst achieved complete conversion of HMF with a selectivity of 60.5% towards FDCA (Table 2, entry 1). In comparison with the monometallic Au catalyst, the Au-M bimetallic catalysts demonstrated superior performances in terms of FDCA selectivity, approaching nearly 100% HMF conversion. However, the selectivity for FDCA varied across different doped metals in the Au-M catalysts. The descending order of selectivity, from highest to lowest, was found to be Au-Ni > Au-Pd > Au-Fe > Au-Cu, resulting in FDCA selectivity of 76.6% (Table 2, entry 2), 71.7% (Table 2, entry 5), 68.5% (Table 2, entry 3), and 60.6% (Table 2, entry 4), respectively. Contrast experiments using MgAlO_x_ as the catalyst (Table 2, entry 6) and a blank experiment without any catalyst (Table 2, entry 7) were also conducted for comparison. Minimal conversion of HMF was observed under these conditions, suggesting that the active sites rely on the presence of Au and Au-M nanoparticles. 

**Scheme 3:**
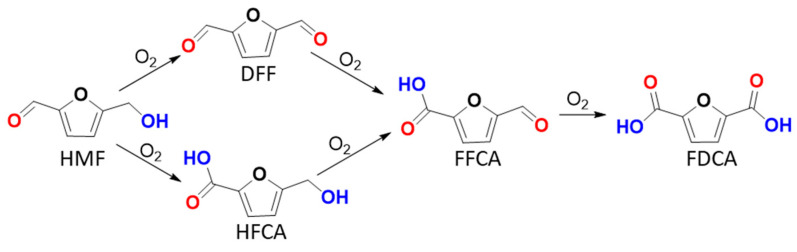
Reaction equation of aerobic oxidation of HMF to FDCA.

**Figure 7:**
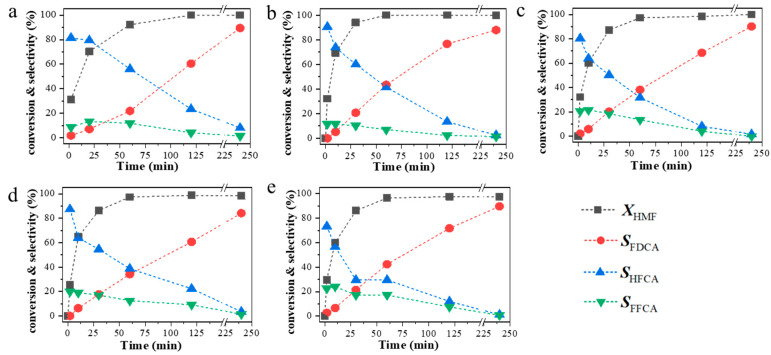
Products distributions over the supported Au and Au-M bimetallic catalysts for aerobic oxidation of HMF: (**a**) Au/MgAlO_x_; (**b**) Au-Ni/MgAlO_x_; (**c**) Au-Fe/MgAlO_x_; (**d**) Au-Cu/MgAlO_x_; and (**e**) Au-Pd/MgAlO_x_. The square represents the conversion of HMF, and the positive triangle, the inverted triangle, and the circle represent the selectivity of HFCA, FFCA, and FDCA, respectively. Reaction conditions: catalyst: 25 mg; C_HMF_: 20 mM; water: 5 mL; molar ratio of HMF/Au: 70; reaction temperature: 90 °C; and pressure of O_2_: 5 atm.

The aerobic oxidation of HMF to FDCA involves a tandem oxidation process that may result in the formation of DFF, HFCA, and FFCA as by-products or intermediates (Scheme 3). In order to monitor the reaction progress, the analysis of product distributions over the above supported Au and Au-M bimetallic catalysts was conducted as a function of reaction time, as shown in Figure 7. The results clearly indicate that within the initial 10 min of the reaction, HFCA is the major product across all catalysts, accompanied by smaller amounts of FDCA and FFCA. By increasing the reaction time, the production of FDCA increases at the expense of HFCA and FFCA. After around 60 min into the reaction, HMF conversions (*X*_HMF_) exceed 90% for all catalysts, with FDCA selectivity (*S*_FDCA_) reaching 21.9% for Au/MgAlO_x_, 43.4% for Au-Ni/MgAlO_x_, 38.2% for Au-Fe/MgAlO_x_, 34.2% for Au-Cu/MgAlO_x_, and 42.3% for Au-Pd/MgAlO_x_ (as shown in Figure 7a–e). Extending the reaction time to 2 h leads to the complete conversion of HMF and increased FDCA selectivity, but the order of the selectivity has not been changed (refer to Table 2). These outcomes strongly indicate that the incorporation of doped metals can enhance catalytic performance, suggesting a synergistic effect between Au and the doped metals. However, further extending the reaction time to 4 h results in the selectivity towards FDCA reaching equilibrium. Specifically, the selectivity percentages for FDCA are 89.4% for Au, 87.9% for Au-Ni, 90.2% for Au-Fe, 84.2% for Au-Cu, and 89.7% for Au-Pd. Importantly, throughout the entire reaction process, the presence of DFF products was not detected in any of the catalysts, which is consistent with our previous findings in the Au-Ag system [40], suggesting that the reaction pathway for HMF tandem oxidation follows the sequence of HMF-HFCA-FFCA-FDCA, as depicted in Scheme 3.

### 2.3. Insight into Improvements to Au-M Bimetallic Catalysts for HMF Oxidation to FDCA

To provide further insight into improvements to Au-M bimetallic catalysts for the oxidation of HMF to FDCA, control experiments over the Au and Au-M bimetallic catalysts for HMF tandem oxidative reactions were conducted. The turnover frequency (TOF) was determined using various substrates, including HMF, DFF, HFCA, and FFCA, to assess the efficiency of the catalysts, which were calculated based on the moles of converted substrates per mole of gold per hour, with all reaction rates maintaining below 20% conversion. Table 3 presents a summary of the obtained TOF values assessed for both Au and Au-M bimetallic catalysts in HMF tandem oxidative reactions. The results reveal varied reaction rates among the evaluated catalysts when exposed to distinct substrates. In terms of HMF oxidation, the Au-Cu/MgAlO_x_ catalyst shows the obvious lowest TOF value (605.0 h^−1^) compared to that of the others (Au: 740.7 h^−1^; Au-Ni: 758.8 h^−1^; Au-Fe: 760.5 h^−1^; Au-Pd: 702.1 h^−1^), indicating the lower efficiency of the f Au-Cu/MgAlO_x_ catalyst in converting the formyl group (-CHO) in HMF. Notably, when DFF was utilized as the reactant, all catalysts exhibited high conversion rates, with complete conversion of DFF achieved within two minutes. This rapid conversion rate suggests that DFF reacts very quickly within the catalysts. Consequently, DFF was undetectable in the process. This result agrees well with our previous data (Table 2). 

Upon further examination of the TOF of different substrates, it becomes evident that HFCA oxidation exhibits significantly lower TOF values. This discrepancy implies that the oxidation of HFCA serves as the rate-determining step in the process. What is noteworthy is that the Au-Ni/MgAlO_x_ catalyst displays the highest TOF value (293.1 h^−1^) for HFCA oxidation compared to other alternative catalysts (Au: 72.9 h^−1^; Au-Fe: 177.5 h^−1^; Au-Cu, 158.2 h^−1^; Au-Pd: 227.2 h^−1^), indicating the superior performance of the Au-Ni/MgAlO_x_ catalyst in HFCA oxidation. In addition, the Au-M bimetallic catalysts exhibit markedly higher TOF values than the monometallic Au catalyst, underscoring that the incorporation of doped metals improves the transformation of the hydroxyl group (−OH) in HFCA conversion. Moreover, a synergistic effect between Au and the doped metals can also be observed in FFCA oxidation, in which the Au-M bimetallic catalysts exhibit higher TOF values than the monometallic Au catalyst (Au-Pd: 883.9 h^−1^; Au-Ni: 671.4 h^−1^; Au-Fe: 704.3 h^−1^; Au-Cu, 675.4 h^−1^; Au: 529.9 h^−1^), demonstrating the easier transformation of the -CHO group in FFCA conversion.

To better comprehend the underlying factors influencing these catalytic performances, Arrhenius plots were conducted on the representative catalysts. The outcomes indicate that the activation energy (Ea) for HMF oxidation is lower over the Au-Fe/MgAlO_x_ catalyst (26 kJ·mol^−1^) than that of the Au-Cu/MgAlO_x_ (31 kJ·mol^−1^) (Figure S3a). Similarly, the Ea for HFCA oxidation is lower for the Au-Ni/MgAlO_x_ (28 kJ·mol^−1^) compared to the Au-Cu/MgAlO_x_ (32 kJ·mol^−1^) (Figure S3b). This result suggests that the incorporation of various doped metals results in distinct catalytic performances during HMF tandem oxidation. In combination, the results of the dynamic tests and the catalytic performances lead to the conclusion that the overall catalytic activity is ranked as follows: Au-Ni > Au-Pd > Au-Fe > Au-Cu > Au. The introduction of Fe and Ni facilitates HMF oxidation and/or HFCA oxidation, while the addition of Pd significantly boosts FFCA oxidation. Although the introduction of Cu diminishes the activity for HMF oxidation, it improves HFCA and FFCA oxidation compared to the monometallic Au catalyst. These findings highlight the varied promoting effects of doped metals on catalytic performances in the tandem oxidative steps. 

**Figure 8:**
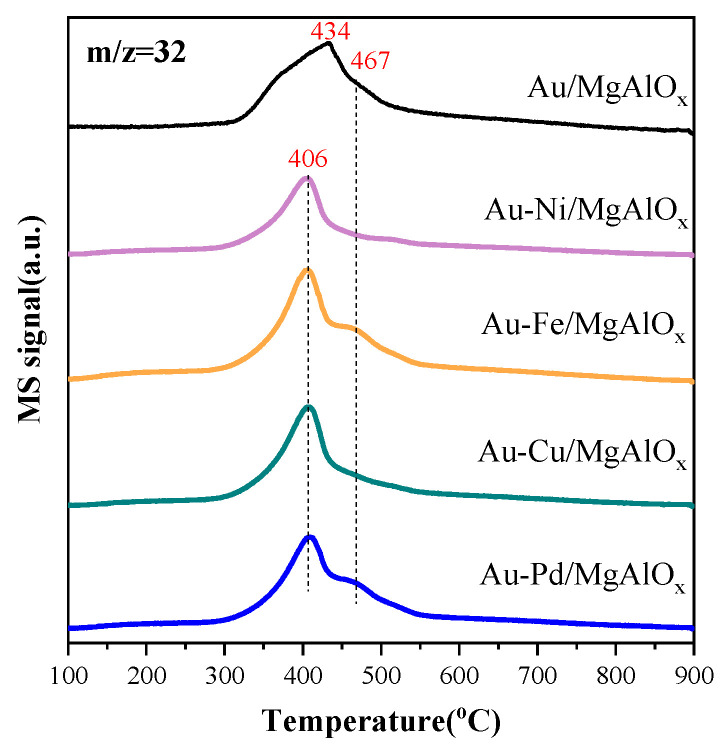
O_2_-TPD profiles of the supported Au and Au–M bimetallic catalysts.

Based on the preceding characterization results, the incorporation of doped metals did not induce any changes in the primary phase of the catalysts, as evidenced in Figure 2 and Figure 3. TEM analyses further confirmed that the Au and Au-M bimetallic catalysts possessed analogous average particle sizes, as depicted in Figure 4. Consequently, it is plausible to infer that the enhancement in the oxidation of HMF by doped metals is independent of particle size considerations. Subsequent examinations via XPS (Figure 5) and in situ CO DRIFTS (Figure 6) revealed that the introduction of secondary metals increases the electron density on Au species, which might potentially facilitate the activation of reactant molecules, thereby enhancing the efficacy of catalysts. Previous research has documented that supported gold catalysts with negatively charged gold species exhibit remarkable catalytic activity in the oxidation of alcohols and glucose [37,57]. Such gold particles, which are rich in electrons, are presumed to activate oxygen molecules by transferring excess charge to the antibonding orbital, resulting in the formation of superoxide or peroxide intermediates [57]. In the present study, the Au-Ni/MgAlO_x_ catalyst, which exhibited the highest electron density, demonstrated superior performance in the HMF tandem reaction process. This outcome is in alignment with previous findings. To further elucidate the activation of oxygen, temperature programmed desorption (TPD) of O_2_ was employed on both monometallic Au and Au–M bimetallic catalysts. As illustrated in Figure 8, a distinct O_2_ desorption peak emerged at 434 °C over the Au/MgAlO_x_ catalyst, which is indicative of the desorption of chemically adsorbed oxygen species on the catalyst surface [58]. Notably, the introduction of doped metals into the catalyst matrix resulted in a reduced O_2_ desorption temperature of 406 °C. This decrease in temperature suggests that the presence of doped metals can elevate the desorption rate of active oxygen species, thereby enhancing the catalytic activity of the Au-M bimetallic catalysts relative to their monometallic Au counterparts.

Although the electronic effects of the second metal on the gold catalyst are considered to contribute to the improved catalytic performance, the role of the base carrier, MgAl-hydrotalcite, in facilitating the dissociation and adsorption of HMF cannot be overlooked [59]. The choice of MgAl-hydrotalcite as the carrier is strategic in avoiding the need for additional inorganic bases, thus maintaining a base-free reaction environment. Although the primary focus of this study lies in the electronic modifications induced by the second metal, the base carrier’s contribution to the catalytic process is recognized and acknowledged.

## 3. Materials and Method

### 3.1. Materials

Chloroauric acid (HAuCl_4_∙4H_2_O, 99.95%, Au > 47.8%) was purchased from Shanghai Jiuyue Chemical Co (Shanghai, China). Copper nitrate (Cu(NO_3_)2·3H_2_O, 99.7%), magnesium nitrate (Mg(NO_3_)2·6H_2_O, 99%), and aluminum nitrate (Al(NO_3_)3·9H_2_O, 99%) were purchased from Sinopharm Chemical Reagent Co., Ltd. (Shanghai, China). Nickel nitrate (Ni(NO_3_)2·6H_2_O, 98%), ferric nitrate (Fe(NO_3_)3·9H_2_O, 99.7%), sodium hydroxide (NaOH, 97%), sodium carbonate (Na2CO3, 99.5%), L-cysteine (Cys, 99%), palladium chloride (PdCl2, 99.9%), 5-hydroxymethylfurfural (HMF, 99%), 2,5-furandicarboxylic acid (FDCA, 98%), 2,5-diformylfuran (DFF, 98%), 5-hydroxymethyl-2-furancarboxylic acid (HFCA, 98%), and 5-formyl-2-furancarboxylic acid (FFCA, 98%) were purchased from Shanghai Aladdin Biochemical Technology Co., Ltd. (Shanghai, China). Sodium borohydride (NaBH4, 97%) was purchased from Shanghai Lingfeng Chemical Reagent Co. (Shanghai, China). All chemicals were used directly without further purification or treatment.

### 3.2. Preparation of the Catalysts

#### 3.2.1. Preparation of the Au NCs and Au-M Bimetallic NCs

Au-based bimetallic NCs were synthesized similarly to monometallic Au NCs, according to our previously reported method, using L-cysteine as the protective ligands [33]. Typically, specific amounts of HAuCl4 (0.1 mmol) and/or metal salt solutions (Ni(NO_3_)2·6H_2_O, Cu(NO_3_)2·3H_2_O, Fe(NO_3_)3·9H_2_O or PdCl_2_, 5 μmol) were dissolved in 20 mL of deionized water and stirred for 5 min. Then under vigorous stirring, certain amounts of the cysteine solution (5.61 mM, 27 mL) were quickly added to the above solution. After 45 min, 3 mL of 1 M NaOH was added to the above solution, followed by adding 2.5 mL of the NaBH4 solution (2 M, 0.2 M NaOH). Finally, the suspension was aged for 3 h at room temperature to obtain the Au and Au-based bimetallic NCs, which were denoted as Au NCs, Au-Cu NCs, Au-Ni NCs, Au-Fe NCs, and Au-Pd NCs.

#### 3.2.2. Preparation of MgAl-Hydrotalcite (HT)

MgAl-HT is synthesized by the coprecipitation method with a Mg/Al molar ratio of 3. Typically, a certain amount of NaOH (17.70 g, 0.44 mol) and Na2CO3 (12.15 g, 0.11 mol) was dissolved in 200 mL of deionized water. Then, a certain amount of Mg(NO_3_)2·6H_2_O (53.88 g, 0.21 mol) and Al(NO_3_)3·9H_2_O (26.37 g, 0.07 mol) was dissolved in 200 mL of deionized water and dropwise added into the above alkaline solution at a reaction rate of 3 mL/min. Under vigorous stirring, the mixture was aged at 75 °C for 24 h. Later, the obtained suspension was filtered and washed with deionized water several times until the pH value of the filtrate was about 7. Finally, the resulting sample was dried overnight under air at 80 °C to obtain MgAl-HT.

#### 3.2.3. Preparation of the Supported Au and Au-M Bimetallic Catalysts

The supported Au and Au-M (M= Ni, Fe, Cu, and Pd) bimetallic catalysts were prepared by the immobilization method. In a typical synthesis, 2 g of Mg-Al hydrotalcite powder was added to the above-obtained solution containing Au or Au-M NCs (Au: 1.9 mM) under vigorous stirring at room temperature. After 2 h, the obtained suspension was filtered, washed, dried at 80 °C overnight, and calcined at 300 °C for 2 h under air with a heating rate of 5 °C/min. The obtained catalysts were denoted as Au/MgAlOx, Au-Ni/MgAlOx, Au-Fe/MgAlOx, Au-Cu/MgAlOx, and Au-Pd/MgAlOx, respectively, with nominal Au loadings of about 1 wt.%.

### 3.3. Catalytic Evaluation of HMF Oxidation

The catalytic oxidation of HMF was evaluated in a batch-type stainless steel autoclave (15 mL) equipped with a magnetic agitator. In a typical test, a suspension solution containing 0.1 mmol of HMF, 25 mg of supported gold catalyst, and 5 mL of water was added to the reactor. After introducing and evacuating O_2_ six times, 5 atm of O_2_ was introduced to the reactor, and the temperature was increased to 90 °C. Then, the reaction mixture was stirred for a fixed time at this condition. After a while, the reactor was quickly removed and cooled to terminate the reaction in an ice bath, and the oxygen was slowly released. The liquid phase products were centrifuged and filtered out to obtain analysis using Agilent Infinity 1260 high-performance liquid chromatography (HPLC) with a photodiode array detector (DAD) and a ChromCore Sugar-10H column (7.8 mm × 300 mm × 6 μm), using a dilute H_2_SO_4_ solution (20 mM in water, flow rate: 0.5 mL/min) as the mobile phase. To ensure the accuracy of the results, all reaction products were measured three times in parallel. Quantitative measurements using the external standard method were based on the calibration of different concentrations of standard products at specific wavelengths (260 nm for HMF, HFCA, and FFCA; 230 nm for FDCA). The conversion of HMF and product selectivity was calculated using Equations (1) and (2):

(1) $$\mathrm{Conversion}\;(\%)=\frac{\mathrm{Moles}\;\mathrm{of}\;\mathrm{reacted}\;\mathrm{HMF}}{\mathrm{Moles}\;\mathrm{of}\;\mathrm{initial}\;\mathrm{HMF}}\;\times \;100\%$$

(2) $$\mathrm{Selectivity}\;(\%)=\frac{\mathrm{Moles}\;\mathrm{of}\;\mathrm{formed}\;\mathrm{product}}{\mathrm{Moles}\;\mathrm{of}\;\mathrm{reacted}\;\mathrm{HMF}}\;\times \;100\%$$

### 3.4. Characterization

UV–visible (UV–vis) spectra were recorded on a TU-1810 spectrometer at room temperature with the transmission mode using water as a reference. A continuous scan between 400 and 900 nm at a scan rate of 100 nm/min was recorded. X-ray diffraction (XRD) analysis was carried out on a PW3040/60 X’Pert PRO (PANalytical, Almelo, The Netherlands) diffractometer, with Cu Kα radiation source (λ = 0.15432 nm) in a certain range of angles (2θ) from 10 to 90°. The actual contents of Au and other metals were analyzed by inductively coupled plasma atomic emission spectroscopy (ICP-AES) on the IRIS Intrepid II XSP instrument (Thermo Electron Corporation), with aqua regia to dissolve the catalysts. The specific surface area, pore volume, and pore size distribution of the catalysts were measured by nitrogen physisorption isotherms on a Quantachrome NT3LX-2 instrument at 77 K, by using the BET and BJH methods for calculation. Notably, all samples were degassed at 300 °C for 120 min prior to analysis. Transmission electron microscopy (TEM) and high-resolution transmission electron microscopy (HRTEM) were acquired on a JEM-2100F microscope at an acceleration voltage of 200 kV. The samples were sonicated, dispersed in ethanol, and dropped onto a copper grid coated with carbon for observation. X-ray photoelectron spectra (XPS) were conducted on an ESCLALAB 250Xi X-ray photoelectron spectrometer by using monochromatic Al Kα radiation (1846.6 eV) as the X-ray source. In situ diffuse reflectance infrared Fourier transform spectroscopy of CO adsorption (CO-DRIFTS) was conducted on a Bruker INVENIO Fourier-transform infrared spectrometer (Karlsruhe, Germany) equipped with a MCT detector in the range of 600–4000 cm^−1^. Prior to the test, the catalyst was treated in a He flow (30 mL/min) at 120 °C for 30 min. Then, the background spectra and desired spectra were recorded at 25 °C under atmospheric pressure in helium. Temperature-programmed desorption of oxygen (O2-TPD) experiments were carried out on a Micromeritics Autochem II 2920 chemisorber, equipped with a thermal conductivity detector (TCD) and mass spectrometry (MS). Prior to the analysis, 100 mg of the sample powders was loaded into a U-type quartz tube reactor and outgassed at 300 °C for 1 h in a He gas flow (50 mL·min^−1^). Afterwards, the samples were cooled down to 100 °C and saturated with a mixture of 10%O_2_/90%He (30 mL·min^−1^) for 2 h. Later, the samples were purged with helium (30 mL·min^−1^) at 100 °C for 30 min. The O_2_-TPD signals were recorded simultaneously using a TCD and MS detector from 100 °C to 900 °C at a heating rate of 10 °C·min^−1^.

## 4. Conclusions

We have successfully synthesized a series of Au-M bimetallic catalysts with controllable sizes for the base-free aerobic oxidation of HMF to FDCA. The reaction pathway for HMF oxidation proceeds via the sequential conversion of HMF to HFCA, FFCA, and finally FDCA. It has been observed that the addition of Fe and Ni promotes the oxidation of HMF and/or HFCA, while the addition of Pd enhances FFCA oxidation. The introduction of Cu diminishes the activity for HMF oxidation but improves HFCA and FFCA oxidation. Consequently, the overall catalytic activity of the catalysts follows the order of Au-Ni > Au-Pd > Au-Fe > Au-Cu > Au. XPS analysis and in situ DRIFTS of CO have been utilized to investigate the electronic properties of the Au-M catalysts. The results demonstrate that the doping of second metals increases the electron density of Au species, thereby facilitating the activation of reactant molecules. This increased electron density is believed to contribute to the enhanced catalytic activity observed in these systems. These findings represent significant advancements in the development of highly efficient Au-based bimetallic catalysts for the aerobic oxidation of biomass-derived platform molecules under base-free conditions. Additionally, they provide valuable insights into the alloying effect in the synthesis of Au-based catalysts.

## Figures and Tables

**Table 1:** Textural properties of the sole support and supported Au-based catalysts.

Entry	Catalysts	Au Loading (%) ^a^	M Loading (%) ^a^	Au/M Molar Ratio	S_BET_ (m^2^/g) ^b^	Total Volume (m^3^/g) ^b^	Pore Size (nm) ^b^
1	MgAlO_x_	-	-	-	79.5	0.33	9.05
2	Au/MgAlO_x_	1.10	-	-	71.5	0.32	9.70
3	Au-Ni/MgAlO_x_	1.09	0.018	18	79.5	0.35	9.07
4	Au-Fe/MgAlO_x_	1.06	0.020	15	89.0	0.39	9.01
5	Au-Cu/MgAlO_x_	1.07	0.022	14	82.6	0.37	9.03
6	Au-Pd/MgAlO_x_	1.08	0.031	19	77.9	0.34	9.09

^a^ As determined by ICP-AES; ^b^ as determined by N_2_ physisorption.

**Table 2:** The catalytic performances of supported Au and Au-based bimetallic catalysts.

Entry	Catalysts	Conversion (%)	Selectivity (%)		
			FDCA	HFCA	FFCA
1	Au/MgAlO_x_	100	60.5	23.5	4.2
2	Au-Ni/MgAlO_x_	100	76.6	13.5	2.9
3	Au-Fe/MgAlO_x_	98.5	68.5	8.1	4.1
4	Au-Cu/MgAlO_x_	98.8	60.6	22.3	9.1
5	Au-Pd/MgAlO_x_	97.6	71.7	12	7.5
6	MgAlO_x_	2.5	0	0	0
7	Blank	0	0	0	0

Reaction conditions: catalyst: 25 mg; C_HMF_: 20 mM; water: 5 mL; Au: 1.4 mol %; reaction temperature: 90 °C; pressure of O_2_: 5 atm; and reaction time: 2 h.

**Table 3:** Control experiments over the Au and Au-M bimetallic catalysts for HMF tandem oxidative reactions.

Entry	Catalysts	TOF (h^−1^) ^a^			
		HMF Oxidation	Dff Oxidation	HFCA Oxidation	FFCA Oxidation
1	Au/MgAlO_x_	740.7	Very fast	72.9	529.9
2	Au-Ni/MgAlO_x_	758.8	Very fast	293.1	671.4
3	Au-Fe/MgAlO_x_	760.5	Very fast	177.5	704.3
4	Au-Cu/MgAlO_x_	605.0	Very fast	158.2	675.4
5	Au-Pd/MgAlO_x_	702.1	Very fast	227.2	883.9

Reaction conditions: catalyst: 15 mg; C_reactant_: 20 mM; water: 5 mL; Au catalysts: 0.84 mol %; reaction temperature: 90 °C; pressure of O_2_: 5 atm; and reaction time: 2 min. ^a^ TOF was calculated by converted substrates per moles of gold per hours, and the conversions were all below 20%.

## Data Availability

Data are available upon request from the corresponding authors.

## Supplementary Materials

The following supporting information can be downloaded at https://www.mdpi.com/article/10.3390/molecules29122724/s1, Figure S1: The XPS survey of different Au-M bimetallic catalysts; Figure S2: (a) Ni 2p; (b) Fe 2p; (c) Cu 2p and (d) Pd 3d XPS spectra of different Au-based bimetallic catalysts; Figure S3: (a) Arrhenius plots for HMF oxidation over the Au-Fe/MgAlOx and Au-Cu/MgAlOx catalysts; (b) Arrhenius plots for HFCA oxidation over the Au-Ni/MgAlOx and Au-Cu/MgAlOx catalysts; Table S1: The surface atomic ratios of different elements in the supported Au and Au-M bimetallic catalysts derived from XPS spectra.
