# Supplementary material for: Au-Based Bimetallic Catalysts for Aerobic Oxidation of 5-Hydroxymethylfurfural to 2,5-Furandicarboxylic Acid under Base-Free Reaction Conditions
Source: Molecules. 2024 Jun 7;29(12):2724. doi: 10.3390/molecules29122724 (PMC11205606; doi:10.3390/molecules29122724)
Supplement: Supplementary file 1 [file molecules-29-02724-s001.zip › molecules-2995771-supplementary.pdf]

# Au-based bimetallic catalysts for aerobic oxidation of 5-hydroxymethylfurfural to 2,5-furandicarboxylic acid under base-free reaction conditions

*Juan Su [1#], Zongyang Liu [1#], Yuan Tan [1,2,*], Yan Xiao [1], Nannan Zhan [1], Yunjie Ding [1,3,4 *]*

[1] *Hangzhou Institute of Advanced studies, Zhejiang Normal University, 1108 Gengwen Road, Hangzhou 311231, China.*

[2] *Key Laboratory of the Ministry of Education for Advanced Catalysis Materials, Zhejiang Normal University, 688 Yingbin Road, Jinhua 321004, China.*

[3] *Dalian National Laboratory for Clean Energy, Dalian Institute of Chemical Physics, Chinese Academy of Sciences, 457 Zhongshan Road, Dalian 116023, China.*

[4] *The State Key Laboratory of Catalysis, Dalian Institute of Chemical Physics, Chinese Academy of Sciences, Dalian 116023, China.*

[*] *Correspondence: yuantan2012@zjnu.edu.cn (Y.T.); dyj@dicp.ac.cn (Y.D.)*

[#] *These authors contributed equally to this work.*

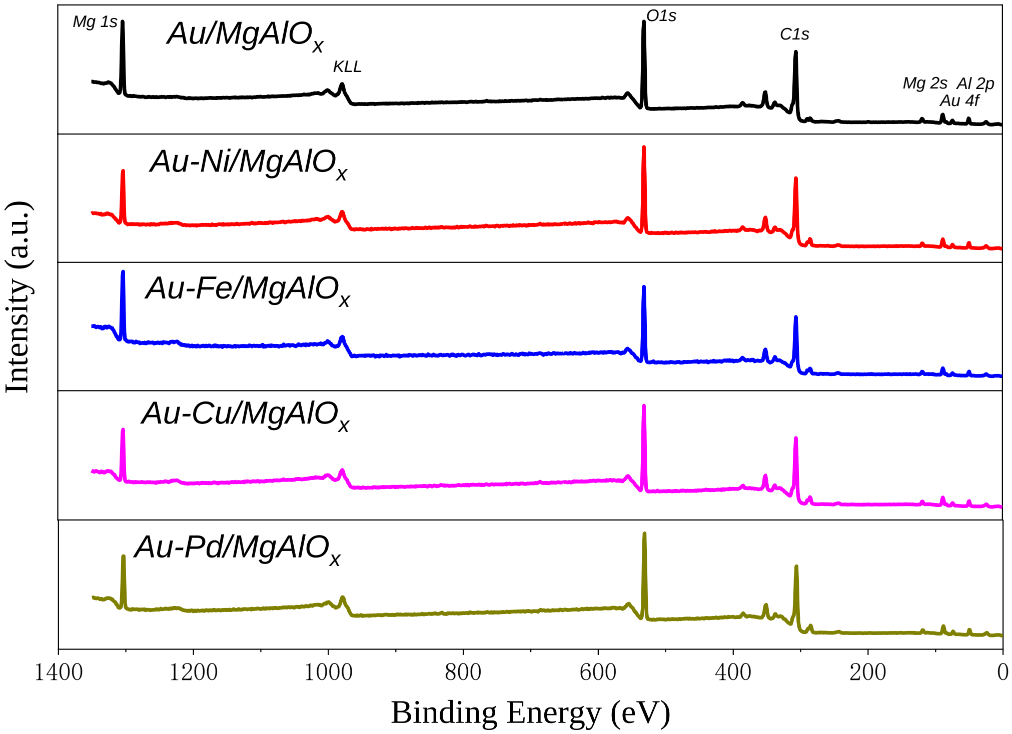


**Figure S1.** The XPS survey of different Au-M bimetallic catalysts.

**Table S1.** The surface atomic ratios of different elements in the supported Au and Au-M bimetallic catalysts derived from XPS spectra.

| Atom% | Au/$MgAlO_x$ | Au-Ni/$MgAlO_x$ | Au-Fe/$MgAlO_x$ | Au-Cu/$MgAlO_x$ | Au-Pd/$MgAlO_x$ |
|---|---|---|---|---|---|
| Mg 1s | 19.19 | 21.0 | 23.65 | 19.39 | 18.88 |
| Al 2p | 9.3 | 7.31 | 7.58 | 8.28 | 7.88 |
| S 2P | 0.25 | 0.23 | 0.36 | 0.23 | 0.27 |
| C 1s | 9.61 | 16.04 | 15.49 | 16.03 | 17.01 |
| O 1s | 60.84 | 54.52 | 51.94 | 55.27 | 54.71 |
| Au 4f | 0.82 | 0.72 | 0.68 | 0.70 | 0.71 |
| M | - | 0.18 | 0.30 | 0.10 | 0.53 |

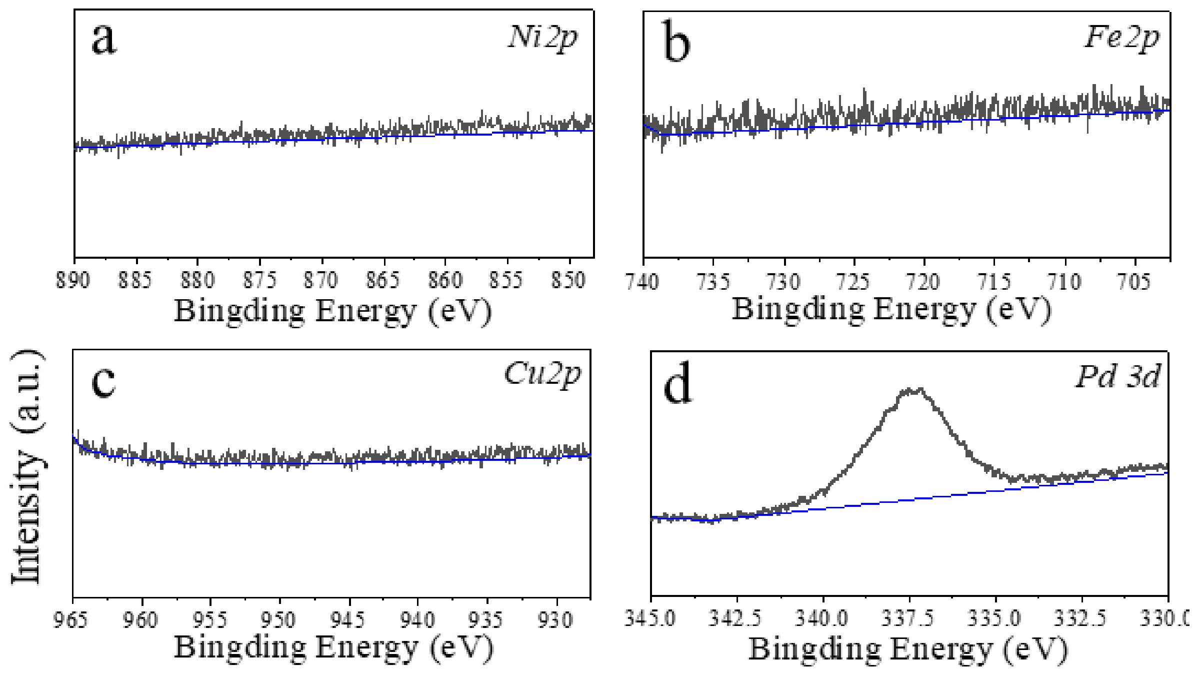


**Figure S2.** (a) Ni 2p; (b) Fe 2p; (c) Cu 2p and (d) Pd 3d XPS spectra of different Au-based bimetallic catalysts.

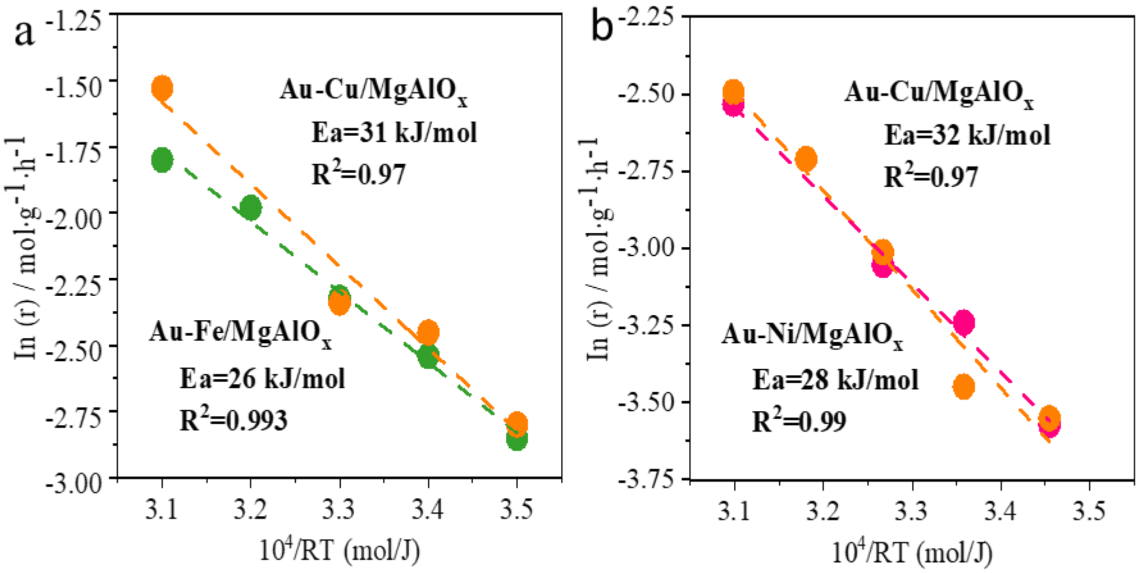


**Figure S3.** (a) Arrhenius plots for HMF oxidation over the Au-Fe/$MgAlO_x$ and Au-Cu/$MgAlO_x$ catalysts, Reaction conditions: catalyst: 15 mg, $C_{HMF}$: 20 mM, water: 5 mL, pressure of $O_2$: 5 atm, reaction time: 1 min, reaction rates (r) were calculated by converted substrates per gram of catalyst per hours, the conversions were all below 20%; (b) Arrhenius plots for HFCA oxidation over the Au-Ni/$MgAlO_x$ and Au-Cu/$MgAlO_x$ catalysts. Reaction conditions: catalyst: 15 mg, $C_{HFCA}$: 20 mM, water: 5 mL, pressure of $O_2$: 5 atm, reaction time: 2 min, r were calculated by converted substrates per gram of catalyst per hours, the conversions were all below 20%.
